# Renal manifestations of severe Rabson-Mendenhall syndrome: a case report

**DOI:** 10.1186/2251-6581-12-7

**Published:** 2013-01-22

**Authors:** Yih Harng Chong, Barry J Taylor, Benjamin J Wheeler

**Affiliations:** 1 Department of Endocrinology, Dunedin Public Hospital, Dunedin, 9054, New Zealand; 2 Department of Women’s and Children’s Health, University of Otago, Dunedin, New Zealand; 3 Edgar National Centre for Diabetes and Obesity Research, University of Otago, Dunedin, New Zealand

**Keywords:** Rabson-Mendenhall syndrome, Insulin resistance, Medullary sponge kidney, Nephrocalcinosis, Hydronephrosis, Nephromegaly

## Abstract

**Introduction:**

Rabson-Mendenhall Syndrome (RMS) is a rare form of severe insulin resistance due to a recessive mutation of the insulin receptor. Associated manifestations include facial dysmorphism, skin abnormalities, and renal anomalies.

**Case presentation:**

We report a case of a 13 year old African female with RMS, severe insulin resistance, and a cluster of renal pathologies including nephromegaly, nephrolithiasis, hydronephrosis, and medullary sponge kidney.

**Conclusion:**

This is the first case of severe insulin resistance associated with the collection of renal conditions described. We postulate that renal conditions present in RMS may be under recognised, and recommend screening for the above conditions. This case adds to the scarce body of literature of associated renal manifestations with RMS, including medullary sponge kidney, across the spectrum of insulin resistance.

## Background

Rabson-Mendenhall Syndrome (RMS) is due to an autosomal recessive mutation of the insulin receptor, with consequential variable insulin resistance. In terms of severity, RMS occupies the intermediate spectrum of the continuum of insulin resistance, being flanked by the milder phenotype of Type A insulin resistance, and on the other side by the more severe condition of Leprechaunism (also known as Donohue Syndrome) [1].The manifestations of severe insulin resistance include hyperinsulinaemia, post prandial hyperglycemia, paradoxical fasting hypoglycaemia, acanthosis nigricans, lipoatrophy, growth retardation, and external genital enlargement. The characteristic facial features include dysmorphic coarse facies with a prognathic jawline, fissured tongue, with dental precocity, hyperplasia and caries. Cutaneous findings include hypertrichosis, skin tags, lichenified skin, and onychauxis. A protuberant abdomen is often described [2,3]. Early published cases commonly reported pineal dysfunction and hypertrophy on autopsy [4,5].

A variety of kidney manifestations have been reported in RMS, including nephrocalcinosis with or without hydronephrosis, and nephromegaly [2,3]. Two cases have been published of associated Medullary Sponge Kidney (MSK) [6,7]. Uncertainty remains as to whether these are independent of, or characteristic features, of RMS.

We present the first case of RMS with severe insulin resistance, and all four previously described kidney manifestations: nephrocalcinosis, hydronephrosis, nephromegaly, and radiologically confirmed severe MSK.

## Case presentation

**Figure 1:**
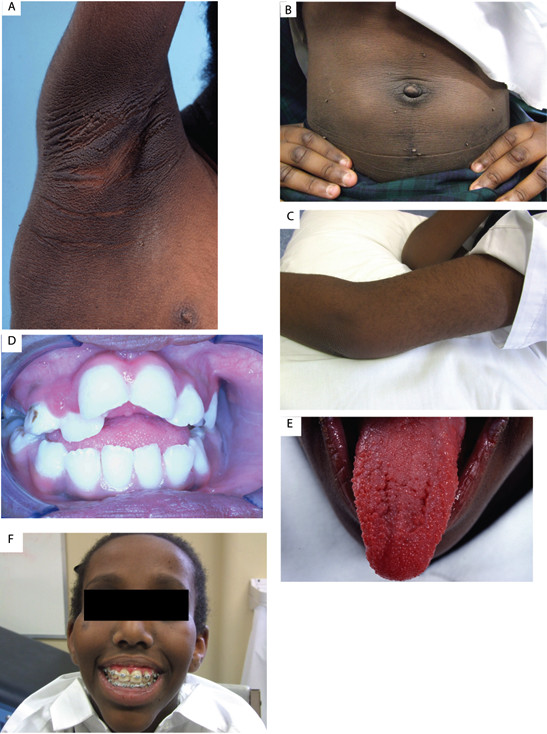
**Clinical manifestations of Rabson Mendenhall Syndrome. A** = Acanthosis Nigricans, **B** = Acrochordons with protruberant abdomen and lipohypertrophy, **C** = hypertrichosis, **D** = Dental dysplasia with caries (younger age), **E** = Fungiform Papillae of Tongue (younger age), **F** = Triangular Facies.

A five year old female presented, shortly after migration from Africa, with chronic scalp infection and otitis media. She was noted to have symptomatic hyperglycaemia, with a history dating back possibly to two years of age. She was observed to have several cutaneous and facial features typical of the RMS phenotype (Figure 1).

Biochemical insulin resistance was documented, with elevated fasting plasma insulin level of 1836 pmol/L (normal laboratory value: 9–80 pmol/L). At age six, diabetes mellitus was diagnosed with the classical pattern of daytime postprandial hyperglycaemia, but paradoxical nocturnal fasting hypoglycaemia. Genetic testing demonstrated a homozygote mutation at position 119 of the mature alpha subunit of the insulin receptor, but a normal beta subunit. She is of African ethnicity, and was born of a consanguineous union between first cousins. There is no other family history of insulin resistance.

Her diabetes was initially managed with Metformin (currently 3 grams/day) followed by a brief trial of added pioglitazone. She rapidly progressed to insulin, with a current insulin requirement of 2100 units per day (>50 units/kg/day). Despite this, her glycaemic control remains suboptimal, with an HbA1c of 75 mmol/mol (9%). She has a current weight of 36 kg, height of 148 cm (Z score −1.1) and BMI of 16.6 kg/m^2^.

Enlarged kidneys were noted from six years of age. Ultrasonography revealed persistent renal sizes greater than two standard deviations for her age (11 cm diameter left, 10 cm diameter right), with poorly defined medullary renal pyramids, loss of normal cortico-medullary differentiation, and a mildly dilated right collecting system. There were no renal cysts or duplex system. Nephrocalcinosis was identified, and appropriate biochemical studies were done, revealing hypercalciuria (Table 1). At age of eight years a mercaptoacetyl triglycine MAG 3 renogram demonstrated normal function bilaterally with an effective renal plasma flow (ERPF) from right kidney of 379 mL/min and left kidney of 404 mL/min (normal ERPF>300 ml/min).

**Table 1:** Biochemical investigations for nephrocalcinosis

**Investigations**	**Results**	**Reference Range**
**Serum:**		
**Calcium**	2.15 mmol / L	2.05 – 2.50 mmol / L
**Phosphate**	1.28 mmol/L	0.9 – 1.65 mmol/L
**Magnesium**	0.79 mmol / L	0.75 – 1.0 mmol / L
**Parathyroid Hormone**	28.3 pg / mL	16 – 87 pg / mL
**25OH Vitamin D**	40 nmol/L	50 – 150 nmol/L
**Creatinine**	30 umol/L	50 – 100 umol/L
**Urine:**		
**24 hour urine calcium excretion**	8.66 mmol/24 hr	1.25 – 6.25 mmol / 24 hr
**Citrate concentration**	5.2 mmol / L	0.4 – 3.4 mmol / L
**Spot urinary cysteine level**	Negative	
**Albumin : Creatinine ratio**	34.1 mg / mmol	< 3.6 mg / mmol

**Figure 2:**
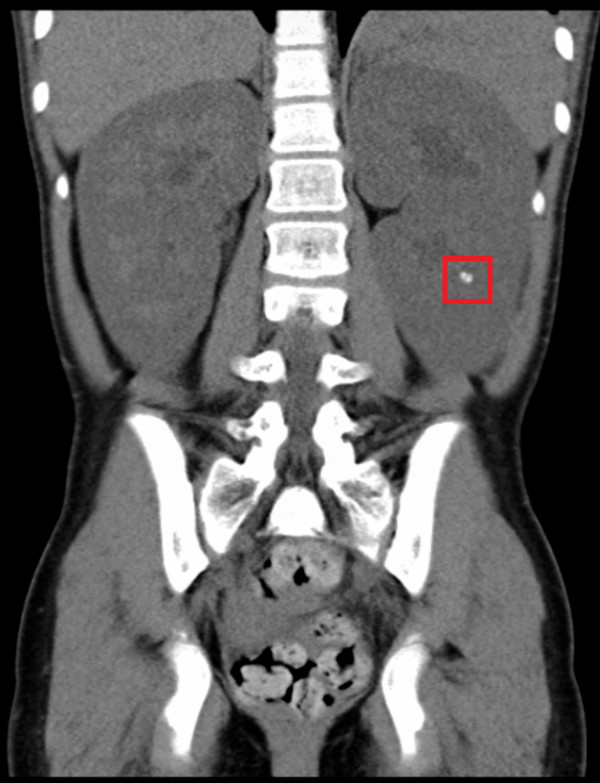
**Renal manifestations on CT abdomen.** Bilateral nephromegaly (Greater than 2 standard deviation for age), and nephrocalcinosis (in red box).

At the age of eleven years she presented with recurrent left flank pain, associated microscopic haematuria, and albuminuria on dipstick examination. There were no features to suggest an infective or traumatic aetiology to her symptoms. She was pre-pubertal. Ultrasonography showed a right kidney of 12.3 cm, left kidney of 13.2 cm, with multiple bilateral renal calculi, the largest 10mm in diameter. The right renal pelvis was dilated (1.2 cm), and a simple 10 mm cyst seen in the left kidney. The bladder was normal. A CT urogram revealed a 2 mm calcification seen to the left of the true renal pelvis, suggestive of an intraluminal ureteric calculus. Intravenous pyelogram further demonstrated paintbrush-like appearances of the renal pyramids, consistent with MSK (Figure 2). The patient had the most severe form of MSK, grade 4, with involvement of all the calyces in both kidneys [8].

**Figure 3:**
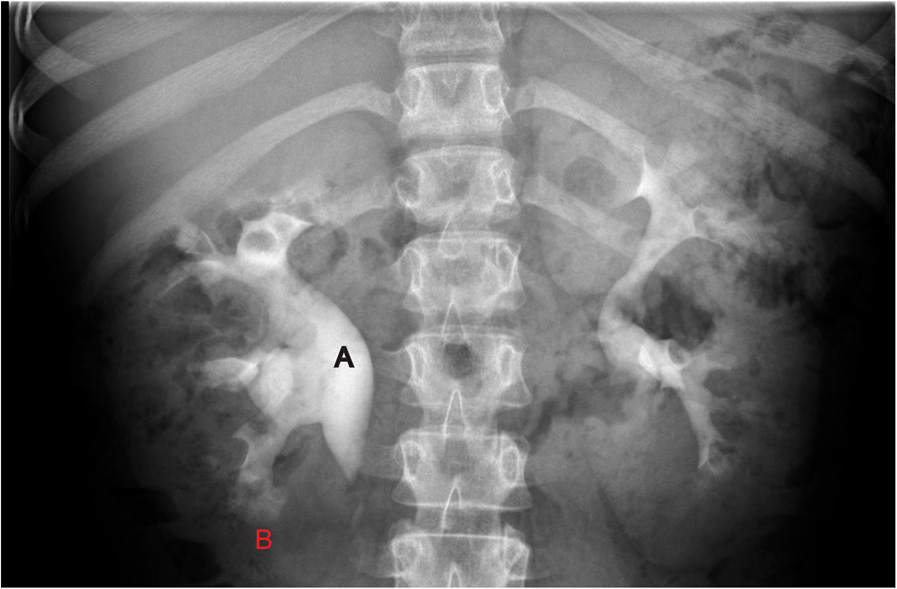
**Renal Manifestations on intravenous urogram. A** = hydronephrosis, **B** = Medullary Sponge Kidney (Paint brush borders in all calyces).

Her nephrocalcinosis was actively managed by optimising fluid intake and a trial of thiazide diuretic. Treatment was ceased at 3 months, at the request of the family as she remained asymptomatic from her nephrocalcinosis. No further acute episodes of nephrocalcinosis or pain have recurred.

## Conclusions

To our knowledge, this is the first case to describe this full constellation of renal manifestations in association with severe insulin resistance and Rabson-Mendenhall syndrome. Given that ours is the third case showing an association of MSK with RMS, we suggest that MSK be considered as part of the RMS, across the full spectrum of insulin resistance. The first such report is described in Harris et al., concerning a 10 year old boy with an incidental finding of MSK during investigation for symptomatic nephrocalcinosis. The main phenotypic difference between this case and ours is the severity of insulin resistance, as the boy required only rosiglitazone and metformin, in contrast to our patient, who needed maximal doses of metformin and very high doses of insulin [6].

Abe et al. reported a case of a Japanese boy with RMS and radiological finding of MSK and nephrocalcinosis, with yet unknown aetiology to link these conditions [7].

Bathi et al. describes a 16 years old female with clinical features of RMS having ultrasonographic evidence of bilateral nephromegaly and hydronephrosis. Unfortunately no mutational analysis was done, and a request for investigation to ascertain any feature of MSK was declined [3]. Other case reports of RMS have either not described any renal features, or only isolated associations, such as medullary nephrocalcinosis [9] or palpable kidneys [10]. We propose that these several renal phenotypes are under-recognised in the RMS population, and we therefore recommend screening for these renal conditions, and especially for nephrocalcinosis.

Furthermore, we demonstrated severe insulin resistance in our patient, with an HbA1c >9% despite a total daily insulin dose of >2000 IU. Given the association in this patient, we postulate that there might be a causal link between hyperinsulinaemia and renal manifestations. Beckwith Wiedemann Syndrome (BWS), a paediatric disorder of excess growth, may present an instructive example. Similar renal manifestations, including nephromegaly, nephrocalcinosis, hydronephrosis, and MSK [11], are seen in BWS. 80% of BWS is associated with a loss of imprinting of chromosome segment 11p15, which encodes for genes including Insulin-like Growth Factor 2 (IGF2) [12]. IGF2 is a potent foetal growth factor, and dysregulation of this hormone can lead to an overgrowth phenotype [12]. Post-partum, IGF2 acts on the IGF1 receptor to exert its anabolic effect. Insulin, which shares homologous structure to IGFs, can bind to IGF1, inducing similar downstream effects [13]. Thus, we may hypothesise that the hyperinsulinaemia in RMS plays a role in the aetiology of the various renal pathologies, via the IGF signalling pathway, although this remains speculative at present [6].

In conclusion, to our knowledge, this is the first case of severe insulin resistance associated with all four renal conditions described, especially with a severe form of MSK. We postulate a mechanism for these renal conditions in RMS. These renal manifestations appear to be under recognised, and screening for the above conditions, in particular nephrocalcinosis, is recommended.

## Consent

Written informed consent was obtained from the patient and the patient’s legal guardian, for publication of this case report and accompanying images. A copy of the written consent is available for review by the Editor-in-Chief of this journal.

## Abbreviations

BWS: Beckwith Wiedemann Syndrome; ERPF: Effective Renal Plasma Flow; IGF: Insulin-like Growth Factor; MSK: Medullary Sponge Kidneys; RMS: Rabson-Mendenhall Syndrome.

## Competing interests

The authors declare that they have no competing interests.

## Authors’ contributions

YC and BW conceived and wrote the manuscript. BT and BW managed the patient from diagnosis through to treatment. BT contributed to writing and editing the manuscript. All authors read and approved the final manuscript.
